# Nutrition and Muscle Health

**DOI:** 10.3390/nu13030797

**Published:** 2021-02-28

**Authors:** Beatrice Arosio, Matteo Cesari

**Affiliations:** 1 Department of Clinical Sciences and Community Health, University of Milan, 20122 Milan, Italy; beatrice.arosio@unimi.it; 2 Geriatric Unit, Fondazione IRCCS Ca’ Granda Ospedale Maggiore Policlinico, 20122 Milan, Italy; 3 Geriatric Unit, IRCCS Istituti Clinici Scientifici Maugeri, 20138 Milan, Italy

The increase in human life expectancy at birth and the rapid aging of the population represent major social phenomena of this time. These changes have enormous implications on public health. In order to promote healthy aging and further increase the years of life spent without disabilities, it is necessary to develop interventions on lifestyle.

A large body of evidence has been produced over the past years to demonstrate the crucial role played by nutrition on the health status of the skeletal muscle. In fact, malnutrition plays a key role in several adverse outcomes in older adults, including chronic disease burden, frailty and mortality. 

In this context, the present Special Issue is timely, advancing the need for successful interventions for preventing physical decline through the promotion of healthy nutrition in older persons. Malnutrition and involuntary weight loss can be driven by anorexia, inadequate dietary intake, sarcopenia, cachexia and/or the combination of these factors. In particular, the anorexia of aging is the driver of unintentional weight loss, mainly affecting the skeletal muscle tissue. Moreover, the energy intake declines at a greater rate than the energy expenditure, causing an imbalance in homeostatic appetite control and, thus, body weight.

In this regard, Johnson et al. [1] investigated the effects of increasing fat-free mass on resting metabolic rate, subjective appetite, appetite-related hormone concentrations and energy intake in older adults through resistance training and protein supplementation. They concluded that chronic resistance training and increased protein intake pose a successful strategy for enhancing fat-free mass and energy intake and breaking the vicious cycle of the reduction of appetite, energy intake and muscle mass in older adults.

Generally, physical performance, muscle mass, and muscle strength were significantly lower in malnourished than in well-nourished subjects. Unexpectedly, after 5 years, these parameters did not necessarily change, as described in the SarcoPhAge study [2].

It should be noted that the tools used to assess malnutrition are not optimized for older persons. Regarding this topic, Lau et al. [3] described the use of the Simplified Nutritional Appetite Questionnaire (SNAQ) utilizing a modified cut-point to define abnormal results of the test. This adjustment has improved the capability of SNAQ to detect anorexia and predict negative outcomes in a cohort composed of cognitively intact and functionally independent community-dwelling adults aged 50 years and older in Singapore [3].

Among the negative outcomes of malnutrition, the individual’s frailty is the most impacting. Moreover, Chew et al. [4] reported that poor nutritional status, dietary intake and muscle health mediated the association between osteosarcopenia (i.e., the coexistence of osteoporosis and sarcopenia) and frailty in community-dwelling Asian older adults [4].

Groenendijk et al. [5] showed that hip fracture patients in geriatric rehabilitation wards living in two Dutch nursing homes were characterized by poor nutritional status, dietary intake and muscle health. Nutritional interventions with special attention to increasing energy and protein intake seemed to prevent recurrent fractures, reduce morbidity and optimize recovery after a hip fracture [5]. Accordingly, Coelho-Junior et al. [6] indicated that adequate protein consumption is an important intervention to manage frailty, emphasizing the importance of setting the optimal distribution of protein across meals and assessing several protein-related parameters (instead of the only amount of protein intake). In fact, neither the total protein intake nor meal distribution appeared to be associated with muscle mass, strength or physical function in a cohort of Danish older adults [7]. 

Interestingly, fiber intake seemed to exert a beneficial effect in older men and women belonging to the cohort of the NU-AGE project, a multi-centre study exploring determinants of healthy aging across five European countries [8]. Indeed, as reviewed by Prokopidis et al. [9], the regulation of protein and fiber consumption may serve to define a gut microbiota profile impacting various metabolic pathways related to sarcopenia and obesity.

A dietary pattern based on white rice, fish and seaweeds was associated with a lower prevalence of low muscle mass in Korean men and women, whereas a higher condiment, vegetables and meats consumption was associated with a higher prevalence of low muscle mass in men [10]. In the same way, Kang et al. [11] demonstrated that a complex of leucine-enriched protein, calcium and vitamin D improved the muscle mass in a cohort of Korean healthy subjects between 50 and 64 years of age. In particular, the vitamin D pathway and vitamin D receptor gene seemed to be responsible for different degrees of fragilization, mainly in women in an Italian cohort composed of very old subjects [12]. 

As Guest Editors for this Special Issue, we thank the Publisher for the support and all the Authors who have joined this timely and needed editorial initiative. We recommend this collection of twelve articles to our readers as a timely addition and important contribution to the field. We are confident that this will spur further discourses and open avenues for further researches into the rapidly evolving and fascinating area of nutrition in healthy aging.
